# Supplementary material for: Microbiota long-term dynamics and prediction of acute graft-versus-host disease in pediatric allogeneic stem cell transplantation
Source: Microbiome. 2021 Jun 28;9:148. doi: 10.1186/s40168-021-01100-2 (PMC8240369; doi:10.1186/s40168-021-01100-2)
Supplement: Supplementary file 2 — Additional file 1. Supplementary Table S1. Patient characteristics. Abbreviations: HLA, human leukocyte antigen; TBI, total body irradiation; CY, Cyclophosphamide; VP16, Etoposide; BU, Busulfan; MEL, Melphalan; GvHD, graft-versus-host disease. Supplementary Table S2. Taxonomy of a subset of LDA clade members and corresponding LDA-coefficients in the gut, oral cavity, and nasal cavity. Supplementary Table S3. Taxonomy of aGvHD predictors within the fecal, oral, and nasal microbiota. ASVs that were significantly predicting aGvHD severity according to the conditional inference tree regression model are highlighted in bold. Of the 50 most important gut ASVs identified by the svmLinear model, 17 were confirmed by Boruta feature selection and are listed here. In the oral and nasal cavities, 26 and 12 ASVs were confirmed by Boruta selection, respectively. Listed in bold are those ASVs with a significant predictive effect on aGvHD severity, tested in a regression framework with CTREE (see Methods). [file 40168_2021_1100_MOESM2_ESM.pdf]

Additional File 1 – Supplementary Tables

Supplementary Table S1. Patient characteristics. Abbreviations: HLA, human leukocyte antigen; TBI, total body irradiation; CY, Cyclophosphamide; VP16, Etoposide; BU, Busulfan; MEL, Melphalan; GvHD, graft-versus-host disease.

**Characteristics**		**Number of patients**	**Percentage of all patients (%)**
**Transplant recipients**		29	100
**Average recipient age in years**		9.5 (Range: 2.5-16.4)	NA
**Gut microbiota characterized**	**At ≤ 6 timepoints**	9	31
	**At 7-8 timepoints**	13	44.8
	**At 9-10 timepoints**	7	24.1
**Oral microbiota chracterized**	**At ≤ 6 timepoints**	3	10.3
	**At 7-8 timepoints**	11	37.9
	**At 9-10 timepoints**	15	51.7
**Nasal microbiota chracterized**	**At ≤ 6 timepoints**	3	10.3
	**At 7-8 timepoints**	9	31
	**At 9-10 timepoints**	17	58.6
**Patient sex**	**Female**	13	44.8
	**Male**	16	55.2
**Disease at transplantation**	**Malignant hematologic diseases**	20	69
	**Severe aplastic anemia**	1	3.4
	**Other benign disorders including immunodeficiencies**	8	27.6
**Donor type**	**HLA-matched sibling**	14	48.3
	**HLA-matched unrelated donor (9/10 or 10/10 match)**	12	41.4
	**Haplo-identical related donor (5/10 match)**	3	10.3
**Stem cell source**	**Bone marrow**	23	79.3
	**Umbilical cord blood**	2	6.9
	**Peripheral blood**	4	13.8
**Conditioning regimen**	**TBI + CY / TBI + VP16**	6	20.7
	**Combinations of BU, CY, VP16 and MEL**	6	20.7
	**Fludarabine-thiothepa combinations**	12	41.4

	**Other combinations with fludarabine**	5	17.2
**Anti-thymocyte globulin treatment**		16	55.2
**Antibiotics pre- and post-HSCT**		29	100
**Sex mismatch (female donor to male recipient)**		7	24.1
**Acute GvHD**	**Grade 0-I**	20	69
	**Grade II-IV**	9	31
**Infections**	**At least one registered bacterial infection**	25	86.2
	**At least one registered viral infection**	29	100
	**At least one registered fungal infection**	14	48.3
**Overall Survival***	**Alive**	26	89.7
	**Dead**	3	10.3
**Relapse of primary disease***		2	6.9
**Non-relapse mortality**		2	6.9
**Re-transplantation**		1	3.4

*Mean follow-up time after HSCT: 21.4 months (range: 10.1 – 32.7 months)

Supplementary Table S2. Taxonomy of a subset of LDA clade members and corresponding LDA-coefficients in the gut, oral cavity, and nasal cavity.

ASV number	Body site	Phylum	Family	Genus/Species/ Description	LDA coefficient (beta)	Number of patients with presence of this ASV at ≥ 1 time point
	**Gut**					
ASV_1	gut	*Firmicutes*	*Enterococcaceae*	*Enterococcus* sp.	0.082	29
ASV_30	gut	*Firmicutes*	*Enterococcaceae*	*Enterococcus* sp.	0.006	26
ASV_158	gut	*Firmicutes*	*Enterococcaceae*	*Enterococcus* sp.	0.006	13
ASV_178	gut	*Firmicutes*	*Enterococcaceae*	*Enterococcus* sp.	0.006	12
ASV_344	gut	*Firmicutes*	*Enterococcaceae*	*Enterococcus* sp.	0.006	8
ASV_395	gut	*Firmicutes*	*Enterococcaceae*	*Enterococcus* sp.	0.012	14
ASV_424	gut	*Firmicutes*	*Enterococcaceae*	*Enterococcus* sp.	0.012	9
ASV_543	gut	*Firmicutes*	*Enterococcaceae*	*Enterococcus* sp.	0.006	7
ASV_552	gut	*Firmicutes*	*Enterococcaceae*	*Enterococcus avium*	0.006	7
ASV_720	gut	*Firmicutes*	*Enterococcaceae*	*Enterococcus* sp.	0.078	15
ASV_730	gut	*Firmicutes*	*Enterococcaceae*	*Enterococcus* sp.	0.006	12
ASV_784	gut	*Firmicutes*	*Enterococcaceae*	*Enterococcus* sp.	0.006	8
ASV_951	gut	*Firmicutes*	*Enterococcaceae*	*Enterococcus* sp.	0.006	12
ASV_1186	gut	*Firmicutes*	*Enterococcaceae*	*Melissococcus* sp.	0.006	9
ASV_3	gut	*Firmicutes*	*Lactobacillaceae*	*Lactobacillus* sp.	0.011	26
ASV_31	gut	*Firmicutes*	*Lactobacillaceae*	*Lactobacillus* sp.	0.006	11
ASV_67	gut	*Firmicutes*	*Lactobacillaceae*	*Lactobacillus sakei*	0.006	15
ASV_74	gut	*Firmicutes*	*Lactobacillaceae*	*Lactobacillus* sp.	0.006	16
ASV_144	gut	*Firmicutes*	*Lactobacillaceae*	*Pediococcus* sp.	0.006	10
ASV_151	gut	*Firmicutes*	*Lactobacillaceae*	*Pediococcus pentosaceus*	0.006	12
ASV_189	gut	*Firmicutes*	*Lactobacillaceae*	*Lactobacillus* sp.	0.006	10
ASV_222	gut	*Firmicutes*	*Lactobacillaceae*	*Lactobacillus rhamnosus*	0.006	8
ASV_586	gut	*Firmicutes*	*Lactobacillaceae*	*Lactobacillus* sp.	0.006	9
ASV_78	gut	*Firmicutes*	*Lachnospiraceae*	*Blautia* sp.	-0.039	26
ASV_237	gut	*Firmicutes*	*Lachnospiraceae*	*Blautia faecis*	-0.026	23
ASV_265	gut	*Firmicutes*	*Lachnospiraceae*	*Blautia* sp.	-0.026	24
ASV_271	gut	*Firmicutes*	*Lachnospiraceae*	*Blautia obeum*	-0.026	12
ASV_375	gut	*Firmicutes*	*Lachnospiraceae*	*Blautia* sp.	-0.026	15
ASV_554	gut	*Firmicutes*	*Lachnospiraceae*	*Blautia* sp.	-0.026	16
ASV_1291	gut	*Firmicutes*	*Lachnospiraceae*	*Blautia* sp.	-0.026	9
ASV_132	gut	*Firmicutes*	*Ruminococcaceae*	*Ruminococcus bromii*	-0.007	16
ASV_192	gut	*Firmicutes*	*Ruminococcaceae*	*Ruminococcus bromii*	-0.007	24
ASV_206	gut	*Firmicutes*	*Ruminococcaceae*	DTU089	-0.024	23
ASV_306	gut	*Firmicutes*	*Ruminococcaceae*	*Ruminococcus* sp.	-0.007	13

ASV_348	gut	*Firmicutes*	*Ruminococcaceae*	*Ruminococcus* sp.	-0.007	11
ASV_556	gut	*Firmicutes*	*Ruminococcaceae*	*Caproiciproducens* sp.	-0.024	9
ASV_566	gut	*Firmicutes*	*Ruminococcaceae*	*Ruminoclostridium sp.*	-0.024	13
ASV_583	gut	*Firmicutes*	*Ruminococcaceae*	*Ruminoclostridium sp.*	-0.024	23
ASV_682	gut	*Firmicutes*	*Ruminococcaceae*	*Caproiciproducens* sp.	-0.024	9
ASV_750	gut	*Firmicutes*	*Ruminococcaceae*	*Caproiciproducens* sp.	-0.024	13
ASV_792	gut	*Firmicutes*	*Ruminococcaceae*	*Ruminoclostridium sp.*	-0.024	15
	**Oral**					
ASV_18	oral	*Actinobacteria*	*Actinomycetaceae*	*Actinomyces* sp.	0.021	27
ASV_66	oral	*Actinobacteria*	*Actinomycetaceae*	*Actinomyces* sp.	0.003	26
ASV_117	oral	*Actinobacteria*	*Actinomycetaceae*	*Actinomyces naeslundii*	0.016	27
ASV_126	oral	*Actinobacteria*	*Actinomycetaceae*	*Actinomyces naeslundii*	0.016	19
ASV_138	oral	*Actinobacteria*	*Actinomycetaceae*	*Actinomyces* sp.	0.003	22
ASV_155	oral	*Actinobacteria*	*Actinomycetaceae*	*Actinomyces odontolyticus*	0.003	20
ASV_227	oral	*Actinobacteria*	*Actinomycetaceae*	*Actinomyces* sp.	0.016	7
ASV_235	oral	*Actinobacteria*	*Actinomycetaceae*	F0332	0.003	18
ASV_262	oral	*Actinobacteria*	*Actinomycetaceae*	*Actinomyces* sp.	0.003	18
ASV_345	oral	*Actinobacteria*	*Actinomycetaceae*	*Actinomyces* sp.	0.016	9
ASV_389	oral	*Actinobacteria*	*Actinomycetaceae*	*Actinomyces odontolyticus*	0.003	7
ASV_403	oral	*Actinobacteria*	*Actinomycetaceae*	*Actinomyces* sp.	0.003	15
ASV_407	oral	*Actinobacteria*	*Actinomycetaceae*	*Actinomyces* sp.	0.016	7
ASV_2693	oral	*Actinobacteria*	*Actinomycetaceae*	*Actinomyces* sp.	0.021	5
ASV_422	oral	*Actinobacteria*	*Actinomycetaceae*	*Actinomyces odontolyticus*	0.003	15
ASV_431	oral	*Actinobacteria*	*Actinomycetaceae*	*Actinomyces* sp.	0.016	12
ASV_2697	oral	*Actinobacteria*	*Actinomycetaceae*	*Actinomyces naeslundii*	0.016	9
ASV_461	oral	*Actinobacteria*	*Actinomycetaceae*	*Actinomyces* sp.	0.003	11
ASV_475	oral	*Actinobacteria*	*Actinomycetaceae*	*Actinomyces gerencseriae*	0.003	6
ASV_484	oral	*Actinobacteria*	*Actinomycetaceae*	*Actinomyces* sp.	0.003	4
ASV_501	oral	*Actinobacteria*	*Actinomycetaceae*	*Actinomyces odontolyticus*	0.003	13
ASV_516	oral	*Actinobacteria*	*Actinomycetaceae*	*Actinomyces odontolyticus*	0.003	12
ASV_2700	oral	*Actinobacteria*	*Actinomycetaceae*	*Actinomyces gerencseriae*	0.003	14
ASV_568	oral	*Actinobacteria*	*Actinomycetaceae*	*Actinomyces* sp.	0.003	12
ASV_600	oral	*Actinobacteria*	*Actinomycetaceae*	*Actinomyces* sp.	0.003	7
ASV_642	oral	*Actinobacteria*	*Actinomycetaceae*	*Actinomyces* sp.	0.003	7
ASV_798	oral	*Actinobacteria*	*Actinomycetaceae*	F0332	0.003	10
ASV_871	oral	*Actinobacteria*	*Actinomycetaceae*	*Actinomyces massiliensis*	0.003	13
ASV_2729	oral	*Actinobacteria*	*Actinomycetaceae*	*Actinomyces graevenitzii*	0.003	9
ASV_1055	oral	*Actinobacteria*	*Actinomycetaceae*	*Actinomyces* sp.	0.003	6

ASV_1172	oral	*Actinobacteria*	*Actinomycetaceae*	*Actinomyces* sp.	0.003	7
ASV_2751	oral	*Actinobacteria*	*Actinomycetaceae*	*Actinomyces* sp.	0.016	8
ASV_2664	oral	*Firmicutes*	*Streptococcaceae*	*Streptococcus* sp.	0.010	29
ASV_10	oral	*Firmicutes*	*Streptococcaceae*	*Streptococcus* sp.	0.010	29
ASV_16	oral	*Firmicutes*	*Streptococcaceae*	*Streptococcus* sp.	0.010	29
ASV_27	oral	*Firmicutes*	*Streptococcaceae*	*Streptococcus* sp.	0.010	29
ASV_28	oral	*Firmicutes*	*Streptococcaceae*	*Streptococcus* sp.	0.040	28
ASV_37	oral	*Firmicutes*	*Streptococcaceae*	*Streptococcus* sp.	0.010	19
ASV_48	oral	*Firmicutes*	*Streptococcaceae*	*Streptococcus* sp.	0.010	27
ASV_173	oral	*Firmicutes*	*Streptococcaceae*	*Streptococcus salivarius*	0.010	15
ASV_183	oral	*Firmicutes*	*Streptococcaceae*	*Streptococcus* sp.	0.010	19
ASV_188	oral	*Firmicutes*	*Streptococcaceae*	*Streptococcus* sp.	0.010	14
ASV_2674	oral	*Firmicutes*	*Streptococcaceae*	*Streptococcus* sp.	0.010	14
ASV_230	oral	*Firmicutes*	*Streptococcaceae*	*Streptococcus* sp.	0.010	8
ASV_269	oral	*Firmicutes*	*Streptococcaceae*	*Streptococcus cristatus*	0.010	29
ASV_282	oral	*Firmicutes*	*Streptococcaceae*	*Streptococcus parasanguinis*	0.010	13
ASV_2683	oral	*Firmicutes*	*Streptococcaceae*	*Streptococcus mitis*	0.010	11
ASV_480	oral	*Firmicutes*	*Streptococcaceae*	*Streptococcus* sp.	0.010	18
ASV_481	oral	*Firmicutes*	*Streptococcaceae*	*Streptococcus peroris*	0.010	12
ASV_802	oral	*Firmicutes*	*Streptococcaceae*	*Streptococcus* sp.	0.010	24
ASV_1531	oral	*Firmicutes*	*Streptococcaceae*	*Streptococcus* sp.	0.010	11
ASV_1599	oral	*Firmicutes*	*Streptococcaceae*	*Streptococcus* sp.	0.010	13
ASV_42	oral	*Bacteroidetes*	*Prevotellaceae*	*Prevotella melaninogenica*	0.028	27
ASV_226	oral	*Bacteroidetes*	*Prevotellaceae*	*Prevotella melaninogenica*	0.028	15
ASV_800	oral	*Bacteroidetes*	*Prevotellaceae*	*Prevotella* sp.	0.028	6
ASV_2665	oral	*Firmicutes*	Family XI	*Gemella* sp.	0.009	29
ASV_208	oral	*Firmicutes*	Family XI	*Gemella sanguinis*	0.009	28
ASV_2701	oral	*Firmicutes*	Family XI	*Gemella* sp.	0.009	5
	**Nasal**					
ASV_14	nasal	*Actinobacteria*	*Corynebacteriaceae*	*Corynebacterium* sp.	0.117	24
ASV_354	nasal	*Actinobacteria*	*Corynebacteriaceae*	*Corynebacterium durum*	0.054	15
ASV_360	nasal	*Actinobacteria*	*Corynebacteriaceae*	*Corynebacterium* sp.	0.054	17
ASV_2704	nasal	*Actinobacteria*	*Corynebacteriaceae*	*Corynebacterium* sp.	0.022	12
ASV_2707	nasal	*Actinobacteria*	*Corynebacteriaceae*	*Corynebacterium durum*	0.054	14
ASV_1206	nasal	*Actinobacteria*	*Corynebacteriaceae*	*Lawsonella* sp.	0.007	16

Supplementary Table S3. Taxonomy of aGvHD predictors within the fecal, oral, and nasal microbiota. ASVs that were significantly predicting aGvHD severity according to the conditional inference tree regression model are highlighted in bold. Of the 50 most important gut ASVs identified by the svmLinear model, 17 were confirmed by Boruta feature selection and are listed here. In the oral and nasal cavities, 26 and 12 ASVs were confirmed by Boruta selection, respectively. Listed in bold are those ASVs with a significant predictive effect on aGvHD severity, tested in a regression framework with CTREE (see Methods).

ASV number	Phylum	Family	Genus/Species/ Description	Body Site	Importance in svmLinear model	Mean Importance in Boruta
	**Gut**					
**ASV_3**	***Firmicutes***	***Lactobacillaceae***	***Lactobacillus sp.***	**gut**	**0.74**	**8.85**
ASV_7	*Bacteroidetes*	*Tannerellaceae*	*Parabacteroides merdae*	gut	0.63	4.62
ASV_8	*Proteobacteria*	*Enterobacteriaceae*	*Escherichia/Shigella* sp.	gut	0.70	4.16
ASV_12	*Firmicutes*	*Ruminococcaceae*	UBA1819 sp.	gut	0.68	5.52
ASV_35	*Bacteroidetes*	*Bacteroidaceae*	*Bacteroides uniformis*	gut	0.68	4.66
ASV_50	*Firmicutes*	*Ruminococcaceae*	*Subdoligranulum* sp.	gut	0.60	3.78
**ASV_128**	***Bacteroidetes***	***Tannerellaceae***	***Parabacteroides distasonis***	**gut**	**0.68**	**7.42**
ASV_131	*Bacteroidetes*	*Tannerellaceae*	*Parabacteroides distasonis*	gut	0.66	6.49
ASV_166	*Fusobacteria*	*Fusobacteriaceae*	*Fusobacterium* sp.	gut	0.60	4.08
**ASV_268**	***Firmicutes***	***Lachnospiraceae***	***Lachnospiraceae_NK4A136_group* sp.**	**gut**	**0.61**	**5.02**
ASV_361	*Bacteroidetes*	*Tannerellaceae*	*Parabacteroides* sp.	gut	0.61	4.40
ASV_477	*Firmicutes*	*Lachnospiraceae*	*Eisenbergiella tayi*	gut	0.62	3.64
ASV_507	*Firmicutes*	*Lachnospiraceae*	*Lachnospira pectinoschiza*	gut	0.60	3.91
ASV_563	*Firmicutes*	*Erysipelotrichaceae*	*Coprobacillus cateniformis*	gut	0.63	5.38
ASV_567	*Firmicutes*	*Ruminococcaceae*	*Flavonifractor* sp.	gut	0.60	2.81
ASV_585	*Bacteroidetes*	*Tannerellaceae*	*Parabacteroides* sp.	gut	0.62	4.53
ASV_687	*Firmicutes*	*Lachnospiraceae*	NA	gut	0.59	3.83
	**Oral**					
ASV_15	*Firmicutes*	*Streptococcaceae*	*Lactococcus* sp.	oral	0.61	7.31
ASV_34	*Proteobacteria*	*Neisseriaceae*	*Neisseria* sp.	oral	0.62	5.08
ASV_66	*Actinobacteria*	*Actinomycetaceae*	*Actinomyces* sp.	oral	0.65	4.84
ASV_72	*Actinobacteria*	*Micrococcaceae*	*Rothia* sp.	oral	0.63	4.49
ASV_92	*Bacteroidetes*	*Bacteroidaceae*	*Bacteroides* sp.	oral	0.65	5.36
ASV_98	*Actinobacteria*	*Micrococcaceae*	*Rothia mucilaginosa*	oral	0.58	5.87
ASV_135	*Firmicutes*	*Streptococcaceae*	*Streptococcus mutans*	oral	0.60	4.24

ASV_205	*Firmicutes*	*Ruminococcaceae*	*Ruminococcaceae_UCG-002* sp.	oral	0.63	4.38
**ASV_226**	***Bacteroidetes***	***Prevotellaceae***	***Prevotella_7 melaninogenica***	**oral**	**0.63**	**8.19**
ASV_247	*Firmicutes*	*Ruminococcaceae*	*Ruminococcaceae_UCG-013* sp.	oral	0.66	4.80
ASV_270	*Firmicutes*	*Veillonellaceae*	*Veillonella* sp.	oral	0.59	3.47
ASV_338	*Bacteroidetes*	*Prevotellaceae*	*Alloprevotella* sp.	oral	0.61	4.26
ASV_360	*Actinobacteria*	*Corynebacteriaceae*	*Corynebacterium* sp.	oral	0.64	6.50
ASV_374	*Fusobacteria*	*Leptotrichiaceae*	*Leptotrichia wadei*	oral	0.60	4.39
ASV_378	*Firmicutes*	*Lachnospiraceae*	*Oribacterium sinus*	oral	0.65	7.17
**ASV_500**	***Actinobacteria***	***Propionibacteriaceae***	***Pseudopropionibacterium propionicum***	**oral**	**0.67**	**8.51**
ASV_518	*Bacteroidetes*	*Bacteroidaceae*	*Bacteroides* sp.	oral	0.64	4.67
**ASV_568**	***Actinobacteria***	***Actinomycetaceae***	***Actinomyces* sp.**	**oral**	**0.71**	**8.79**
ASV_640	*Bacteroidetes*	*Tannerellaceae*	*Parabacteroides* sp.	oral	0.62	2.81
ASV_871	*Actinobacteria*	*Actinomycetaceae*	*Actinomyces massiliensis*	oral	0.61	4.65
ASV_894	*Firmicutes*	*Ruminococcaceae*	*Ruminococcaceae_UCG-014* sp.	oral	0.62	2.74
ASV_1403	*Firmicutes*	*Veillonellaceae*	*Selenomonas_3* sp.	oral	0.59	2.82
ASV_2664	*Firmicutes*	*Streptococcaceae*	*Streptococcus* sp.	oral	0.68	6.11
ASV_2666	*Actinobacteria*	*Micrococcaceae*	*Rothia dentocariosa*	oral	0.61	4.47
ASV_2676	*Firmicutes*	*Aerococcaceae*	*Abiotrophia defectiva*	oral	0.61	5.74
ASV_2692	*Actinobacteria*	*Micrococcaceae*	*Rothia* sp.	oral	0.62	6.30
	**Nasal**					
ASV_2664	*Firmicutes*	*Streptococcaceae*	*Streptococcus* sp.	nasal	0.61	11.49
ASV_2666	*Actinobacteria*	*Micrococcaceae*	*Rothia dentocariosa*	nasal	0.56	5.80
**ASV_47**	***Actinobacteria***	***Micrococcaceae***	***Rothia* sp.**	**nasal**	**0.59**	**6.41**
ASV_51	*Proteobacteria*	*Pasteurellaceae*	*Haemophilus* sp.	nasal	0.64	5.67
ASV_52	*Actinobacteria*	*Micrococcaceae*	*Rothia mucilaginosa*	nasal	0.61	8.58
**ASV_66**	***Actinobacteria***	***Actinomycetaceae***	***Actinomyces* sp.**	**nasal**	**0.67**	**9.05**
ASV_2670	*Proteobacteria*	*Neisseriaceae*	NA	nasal	0.61	6.69
ASV_117	*Actinobacteria*	*Actinomycetaceae*	*Actinomyces naeslundii*	nasal	0.58	4.73
ASV_125	*Actinobacteria*	*Corynebacteriaceae*	*Corynebacterium_1 accolens*	nasal	0.57	5.43
ASV_252	*Firmicutes*	*Veillonellaceae*	*Veillonella rogosae*	nasal	0.58	5.54
ASV_270	*Firmicutes*	*Veillonellaceae*	*Veillonella* sp.	nasal	0.57	5.41

ASV_2694	*Actinobacteria*	*Corynebacteriaceae*	*Lawsonella* sp.	nasal	0.65	8.42
