# Supplementary material for: Microbiota long-term dynamics and prediction of acute graft-versus-host disease in pediatric allogeneic stem cell transplantation
Source: Microbiome. 2021 Jun 28;9:148. doi: 10.1186/s40168-021-01100-2 (PMC8240369; doi:10.1186/s40168-021-01100-2)
Supplement: Supplementary file 3 — Additional file 2 Figure S1. The gut microbiota in the HSCT patients at pre-exam differs from the gut microbiota of age-matched healthy children. A) Fecal bacterial alpha diversity (inverse Simpson index) was 2.4-fold higher in healthy children (n=18) compared to children at pre-examination before HSCT (n=15). B) Fecal bacterial composition was significantly different between the two groups (anosim, p=0.001, R=0.44), and within-group variance was significantly greater in the HSCT group (betadisper, p<0.001). C) The taxa which best explain differences in community structure between HSCT patients at preexamination and healthy children were identified by analysis of LEfSe (Linear discriminant analysis Effect Size). LefSe accounts for the hierarchical structure of bacterial phylogeny, thereby allowing identification of differentially abundant taxa on several taxonomic levels (here: kingdom to genus). Count data was centered-log ratio (CLR) transformed within the LEfSe analysis. The higher the LDA score (log10), the higher the effect size of the respective taxon in explaining group difference. Here, we show taxa with an LDA score >4. D) Differentially abundant genera between the two groups were additionally identified by DESeq2. Of the top 100 most abundant genera (of the whole gut microbiota data set), eighteen genera were significantly more abundant in healthy children (yellow), and 15 genera were significantly more abundant in the patients at preexam (purple). Differences in median proportions of these genera (and their supertaxa) are displayed in a heat tree. See also additional information at https://doi.org/10.6084/m9.figshare.13614230. Figure S2. Most abundant taxonomic families in the gut, oral cavity, and nasal cavity in allo-HSCT patients. Rank abundance curves displaying the proportions of the 12 most abundant taxonomic families at each body site (gut, oral cavity, and nasal cavity). Figure S3. Tree-based sparse linear discriminant analysis revealing nasal ASV [file 40168_2021_1100_MOESM3_ESM.pdf]

Additional File 2 – Supplementary Figures

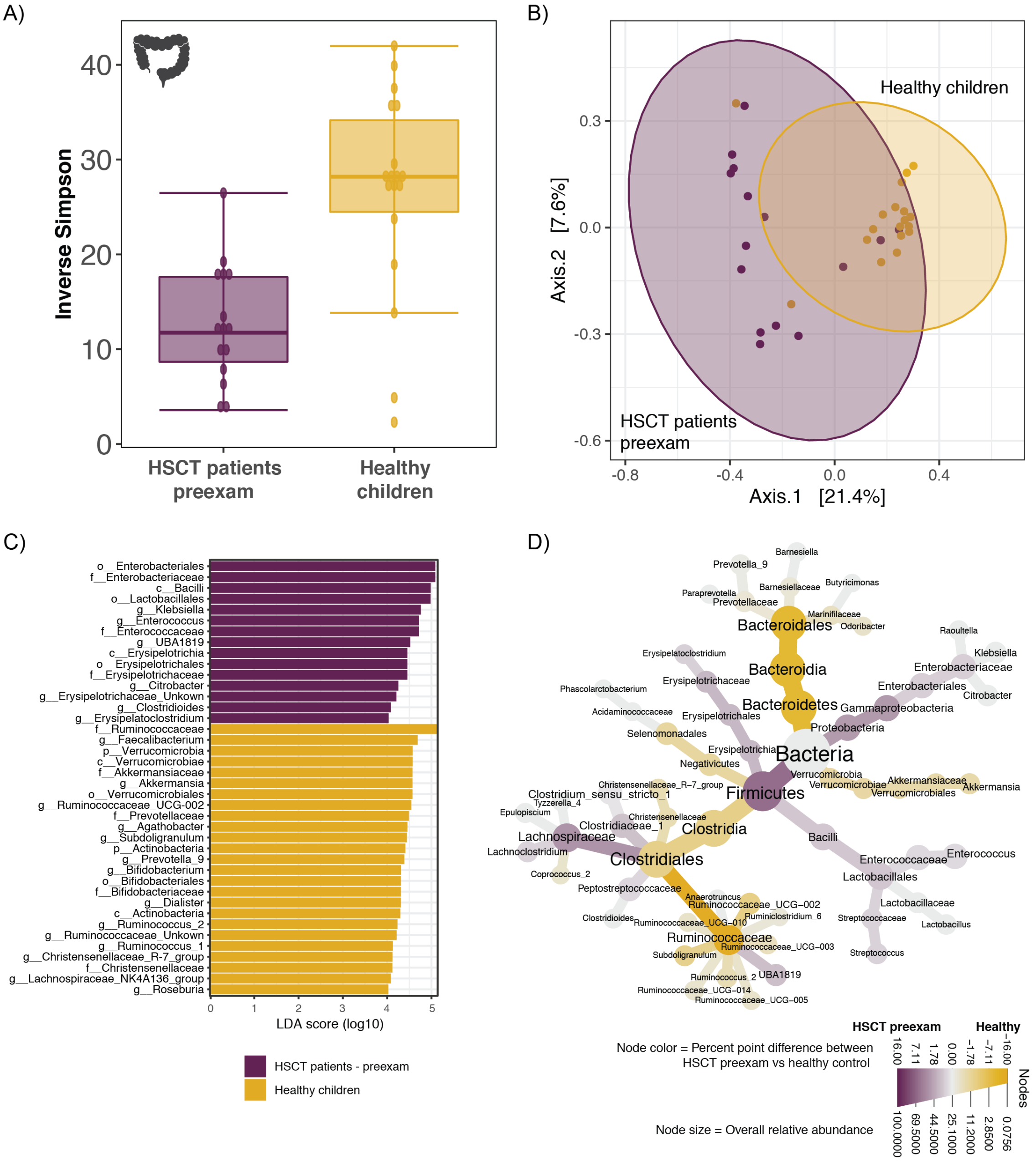

Figure S1. The gut microbiota in the HSCT patients at pre-exam differs from the gut microbiota of age-matched healthy children. A) Fecal bacterial alpha diversity (inverse Simpson index) was 2.4-fold higher in healthy children (n=18) compared to children at pre-examination before HSCT (n=15). B) Fecal bacterial composition was significantly different between the two groups (anosim, p=0.001, R=0.44), and within-group variance was significantly greater in the HSCT group (betadisper, p<0.001). C) The taxa which best explain differences in community structure between HSCT patients at preexamination and healthy children were identified by analysis of LEfSe (Linear discriminant analysis Effect Size). LefSe accounts for the hierarchical structure of bacterial phylogeny, thereby allowing identification of differentially abundant taxa on several taxonomic levels (here: kingdom to genus). Count data was centered-log ratio (CLR) transformed within the LEfSe analysis. The higher the LDA score (log10), the higher the effect

size of the respective taxon in explaining group difference. Here, we show taxa with an LDA score >4. D) Differentially abundant genera between the two groups were additionally identified by DESeq2. Of the top 100 most abundant genera (of the whole gut microbiota data set), eighteen genera were significantly more abundant in healthy children (yellow), and 15 genera were significantly more abundant in the patients at preexam (purple). Differences in median proportions of these genera (and their supertaxa) are displayed in a heat tree. See also additional information at https://doi.org/10.6084/m9.figshare.13614230.

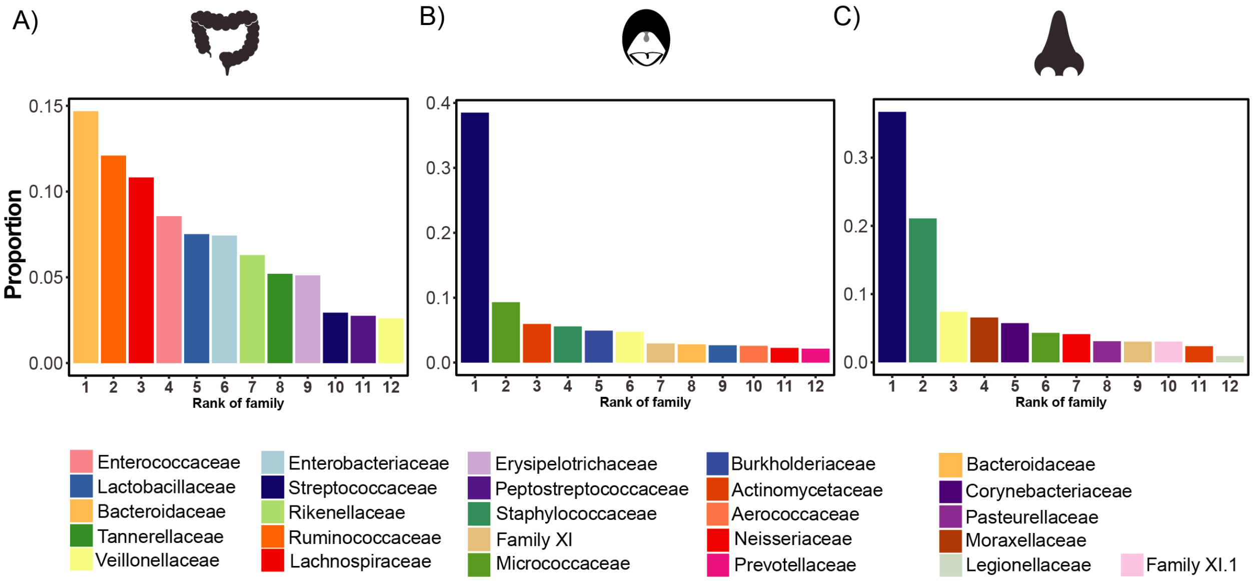

Figure S2. Most abundant taxonomic families in the gut, oral cavity, and nasal cavity in allo-HSCT patients. Rank abundance curves displaying the proportions of the 12 most abundant taxonomic families at each body site (gut, oral cavity, and nasal cavity).

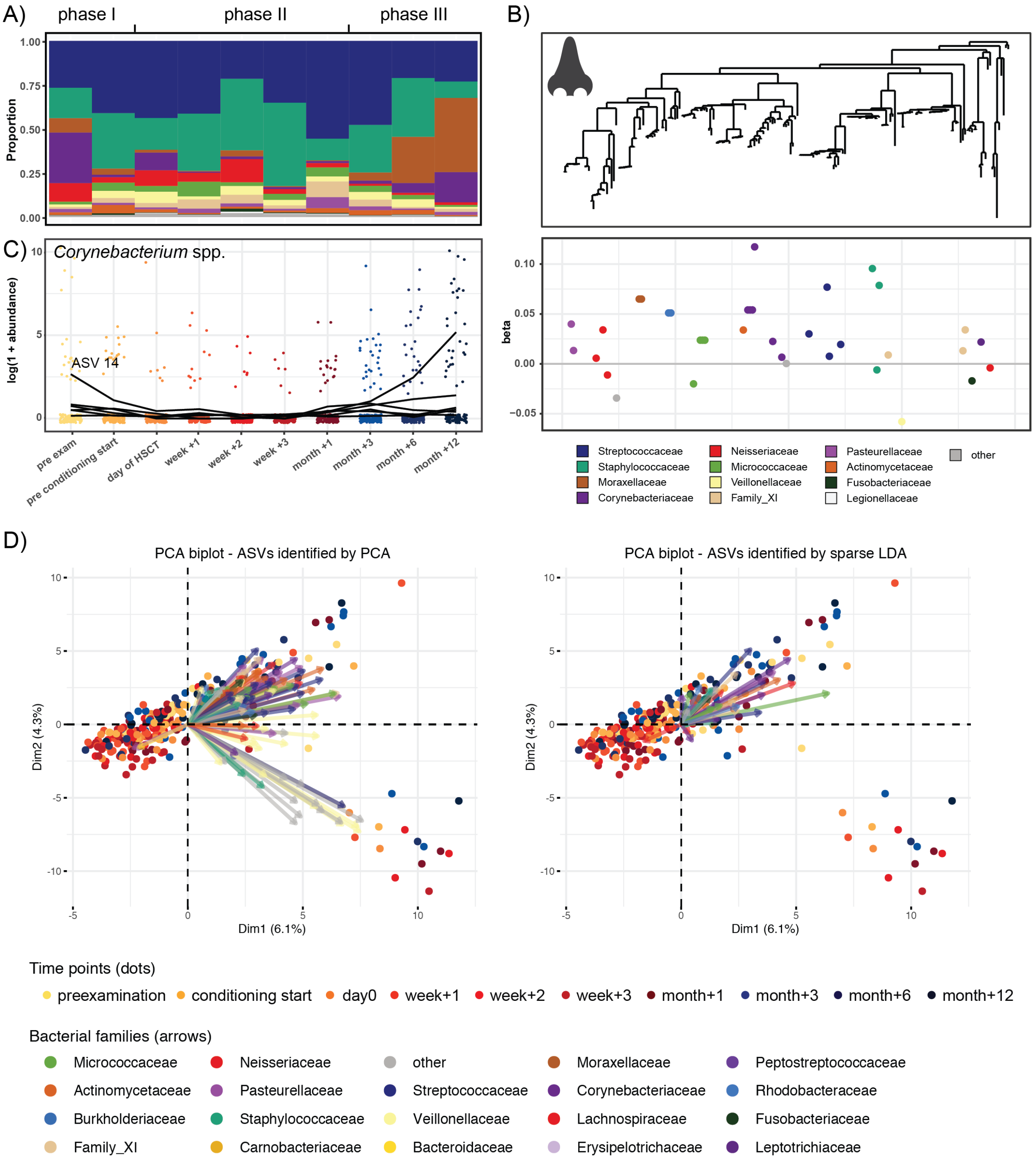

Figure S3. Tree-based sparse linear discriminant analysis revealing nasal ASVs that distinguish time points from each other in relation to HSCT. A) Relative abundances over time of the 12 most abundant families in the nasal cavity. B) Coefficients of discriminating clades of ASVs on the first LDA axis, colored by taxonomic family, and plotted along the phylogenetic tree. C) Trajectories of ASVs in one discriminating group, affiliated with the family *Corynebacteriaceae,* with decreasing abundances after HSCT and recovery at late follow-up time points. The most abundant discriminating ASV is indicated. Detailed taxonomic information and LDA-coefficients of the displayed ASVs are listed in Table S2. D) PCA-biplots with the top 100 predictors (ASVs) identified by PCA (left) and the top predictors (ASVs) identified by sparse LDA (right). The time points are indicated in the same color as in Figure 1C

(phase I: yellow colors; phase II: red colors; phase III: blue colors). The PCA plots with dimensions 1 and 2 are displayed here and additional PCA plots are available from https://doi.org/10.6084/m9.figshare.14510661.

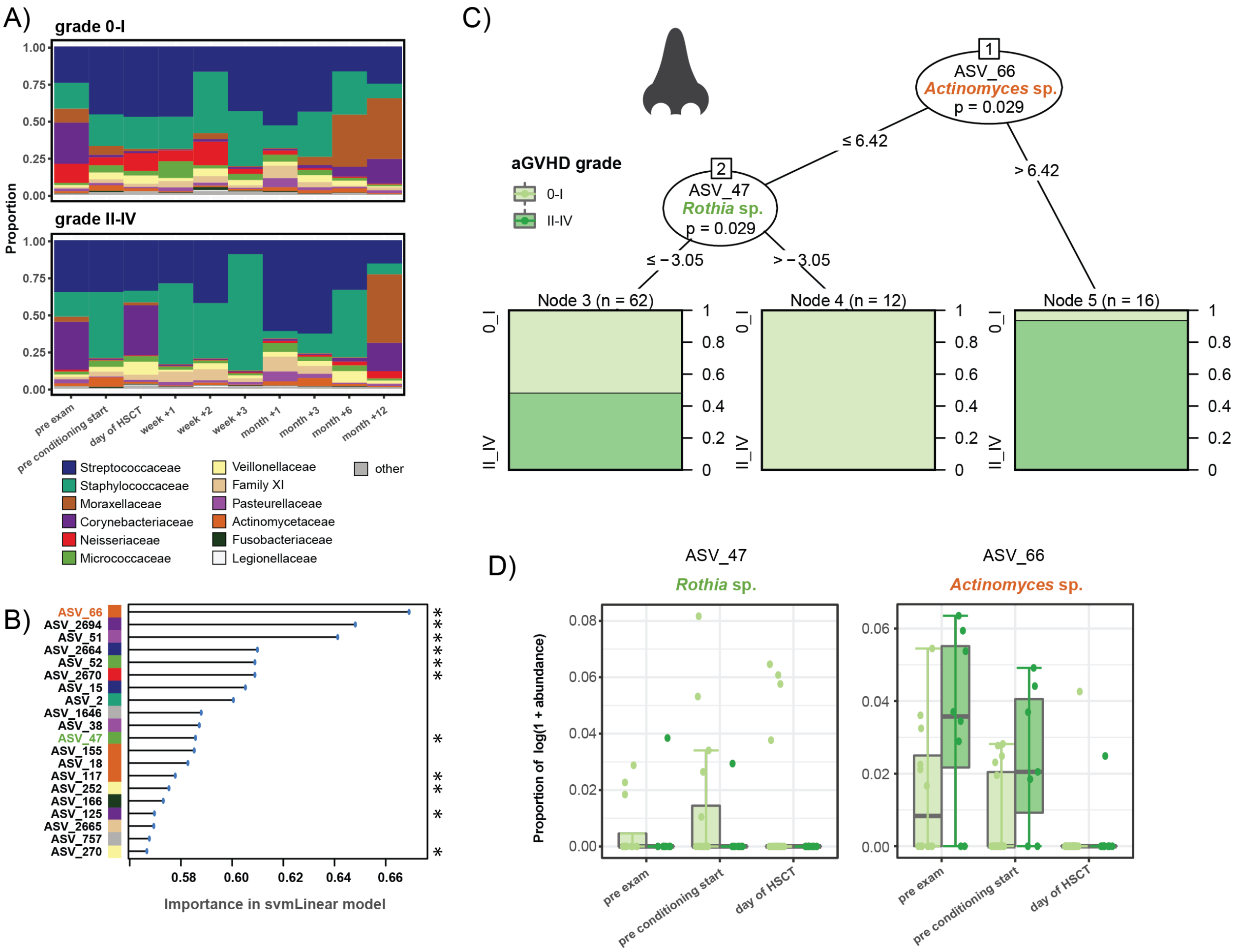

Figure S4. Machine learning-based prediction of aGvHD severity from nasal microbial abundances pre-HSCT. A) Relative abundances of the 12 most abundant families over time in the nasal cavity in patients with aGvHD grade 0-I versus II-IV. B) Importance plot of top 20 predictive nasal ASVs identified by the svmLinear model with importance scores indicating the mean decrease in prediction accuracy in case the respective ASV would be excluded from the model. The final cross-validated svmLinear model predicted aGvHD (0-I versus II-IV) from the abundances of nasal ASVs pre-HSCT with 76% accuracy (95% CI: 56% to 90%). The ASVs that were also confirmed by Boruta feature selection are indicated with asterisk. C) Conditional inference tree (CTREE) displaying ASVs identified as significant split nodes by nonparametric regression for prediction of aGvHD. Numbers along the branches indicate split values of variance stabilized bacterial abundances. The terminal nodes show the proportion of samples originating from patients with aGvHD grade 0-I vs II-IV (n = number of samples). D) Boxplots depict the log transformed relative abundances of the predictive ASVs at time points up to the transplantation in aGvHD grade 0-I compared with grade II-IV patients.

A)

B)

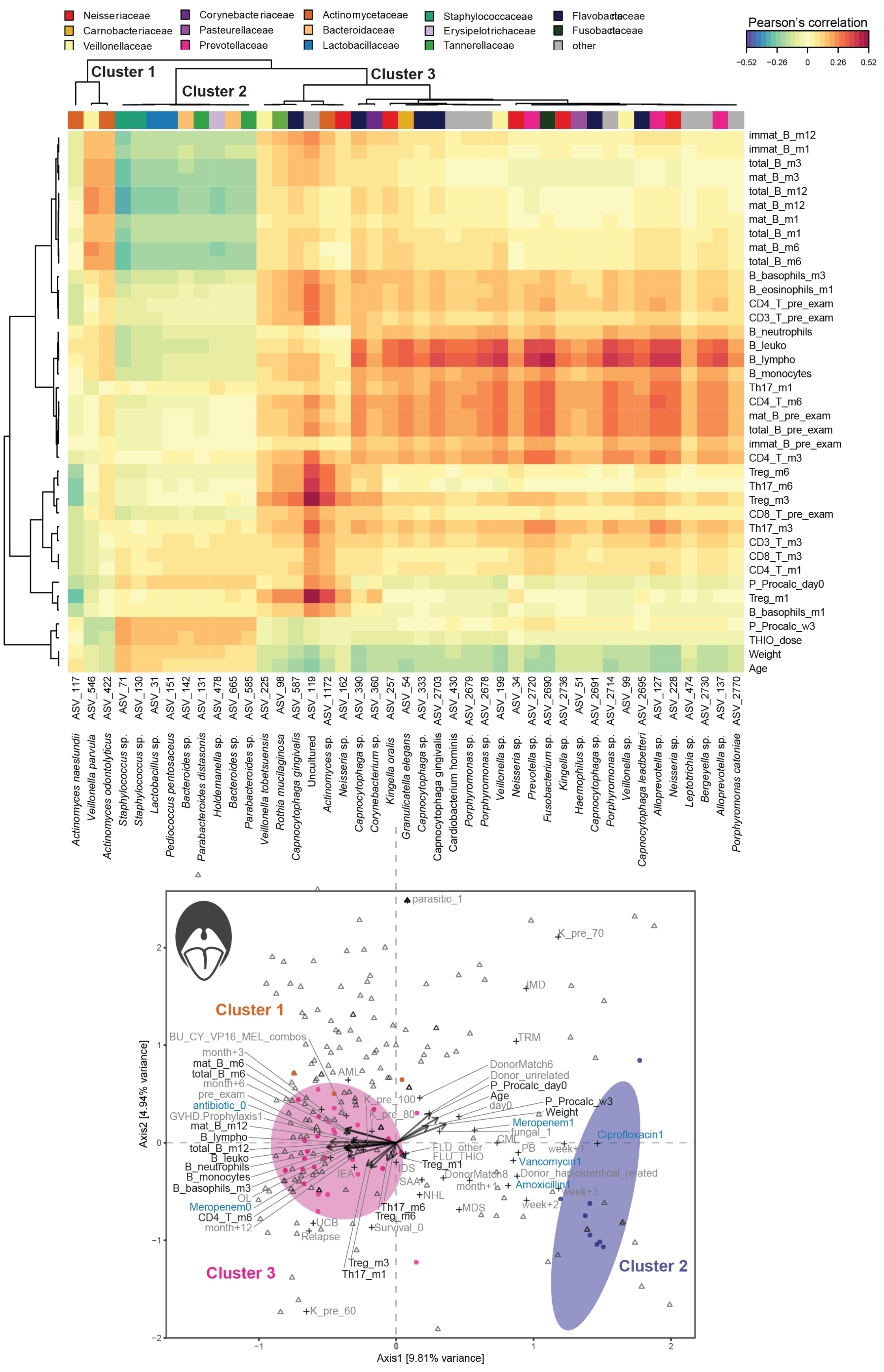

Neisseriaceae
Carnobacteriaceae
Veillonellaceae
Corynebacteriaceae
Pasteurellaceae
Prevotellaceae
Actinomycetaceae
Bacteroidaceae
Lactobacillaceae
Staphylococcaceae
Erysipelotrichaceae
Tannerellaceae
Flavobacteriaceae
Fusobacteriaceae
other
Pearson's correlation
−0.52
−0.26
0
0.26
0.52
Cluster 1
Cluster 2
Cluster 3
immat_B_m12
immat_B_m1
total_B_m3
mat_B_m3
total_B_m12
mat_B_m12
mat_B_m1
total_B_m1
mat_B_m6
total_B_m6
B_basophils_m3
B_eosinophils_m1
CD4_T_pre_exam
CD3_T_pre_exam
B_neutrophils
B_leuko
B_lympho
B_monocytes
Th17_m1
CD4_T_m6
mat_B_pre_exam
total_B_pre_exam
immat_B_pre_exam
CD4_T_m3
Treg_m6
Th17_m6
Treg_m3
CD8_T_pre_exam
Th17_m3
CD3_T_m3
CD8_T_m3
CD4_T_m1
P_Procalc_day0
Treg_m1
B_basophils_m1
P_Procalc_w3
THIO_dose
Weight
Age
ASV_117 Actinomyces naeslundii
ASV_546 Veillonella parvula
ASV_422 Actinomyces odontolyticus
ASV_71 Staphylococcus sp.
ASV_130 Staphylococcus sp.
ASV_31 Lactobacillus sp.
ASV_151 Pediococcus pentosaceus
ASV_142 Bacteroides sp.
ASV_131 Parabacteroides distasonis
ASV_478 Holdemanella sp.
ASV_665 Bacteroides sp.
ASV_585 Parabacteroides sp.
ASV_225 Veillonella tobetsuensis
ASV_98 Rothia mucilaginosa
ASV_587 Capnocytophaga gingivalis
ASV_119 Uncultured
ASV_1172 Actinomyces sp.
ASV_162 Neisseria sp.
ASV_390 Capnocytophaga sp.
ASV_360 Corynebacterium sp.
ASV_257 Kingella oralis
ASV_54 Granulicatella elegans
ASV_333 Capnocytophaga sp.
ASV_2703 Capnocytophaga gingivalis
ASV_430 Cardiobacterium hominis
ASV_2679 Porphyromonas sp.
ASV_2678 Porphyromonas sp.
ASV_199 Veillonella sp.
ASV_34 Neisseria sp.
ASV_2720 Prevotella sp.
ASV_2690 Fusobacterium sp.
ASV_2736 Kingella sp.
ASV_51 Haemophilus sp.
ASV_2691 Capnocytophaga sp.
ASV_2714 Porphyromonas sp.
ASV_99 Veillonella sp.
ASV_2695 Capnocytophaga leadbetteri
ASV_127 Alloprevotella sp.
ASV_228 Neisseria sp.
ASV_474 Leptotrichia sp.
ASV_2730 Bergeyella sp.
ASV_137 Alloprevotella sp.
ASV_2770 Porphyromonas catoniae
parasitic_1
K_pre_70
IMD
TRM
Cluster 1
BU_CY_VP16_MEL_combos
month+3
mat_B_m6
total_B_m6
month+6
pre_exam
antibiotic_0
GVHD.Prophylaxis1
mat_B_m12
B_lympho
total_B_m12
B_leuko
B_neutrophils
B_monocytes
B_basophils_m3
OL
Meropenem0
CD4_T_m6
month+12
AML
K_pre_100
K_pre_80
DonorMatch6
Donor_unrelated
P_Procalc_day0
Age
day0
P_Procalc_w3
Weight
Meropenem1
fungal_1
CML
Ciprofloxacin1
FLU_other
FLU_THIO
PB
week+1
Treg_m1
Vancomycin1
IDS
DonorMatch8
Donor_haploidentical_related
SAA
month+1
Amoxicillin1
IEA
NHL
week+3
week+2
Th17_m6
MDS
Treg_m6
UCB
Survival_0
Relapse
Treg_m3
Th17_m1
Cluster 3
Cluster 2
K_pre_60
Axis2 [4.94% variance]
Axis1 [9.81% variance]
−2
−1
0
1
2

Figure S5. Multivariate associations of the oral microbiota with immune and clinical parameters in HSCT. A) Clustered image map (CIM) based on sparse partial least squares (sPLS) regression analysis dimensions 1, 2, and 3, displaying pairwise correlations >0.2/<-0.2 between oral ASVs (bottom), and continuous immune and clinical parameters (right). Red indicated positive correlation, and blue indicates negative correlation, respectively. Based on the sPLS regression model, hierarchical clustering (clustering method: complete linkage, distance method: Pearson's correlation) was performed resulting in the three depicted clusters. B) Canonical correspondence analysis (CCpnA) relating oral microbial abundances (circles) to continuous (arrows) and categorical (+) immune and clinical parameters. ASVs and variables with at least one correlation >0.2/<-0.2 in the sPLS analysis were included in the CCpnA. The triplot shows variables and ASVs with a score >0.3/<-0.3 on at least one the first three CCpnA axes, displayed on axis 1 versus 2 with samples depicted as triangles. The colored ellipses (depicted with 80% confidence interval) correspond to the clusters of ASVs identified by the sPLS-based hierarchical clustering. For visualization purposes, a focused section of the CCpnA triplot is shown. Antibiotics are indicated in blue font color. Abbreviations are described in Figure 6. Additional abbreviations: fungal, fungal infection; haploident, haploidentical donor; hemo, hemoglobin; leuko, leukocytes; lympho, lymphocytes; w1, week+1; w2, week+2; w3, week+3.

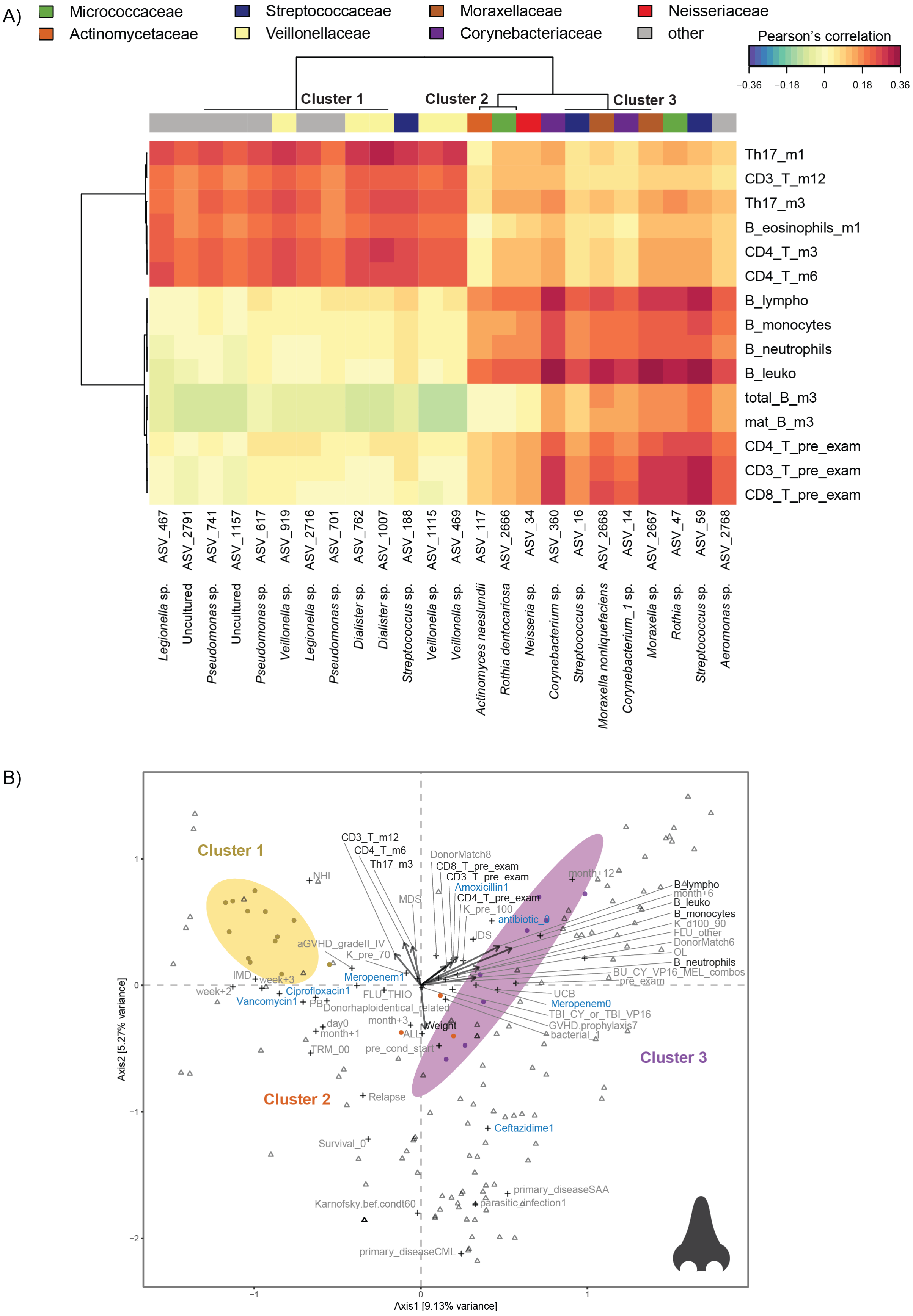

A)
Micrococcaceae
Actinomycetaceae
Streptococcaceae
Veillonellaceae
Moraxellaceae
Corynebacteriaceae
Neisseriaceae
other
Pearson's correlation
−0.36 −0.18 0 0.18 0.36
Cluster 1
Cluster 2
Cluster 3
Th17_m1
CD3_T_m12
Th17_m3
B_eosinophils_m1
CD4_T_m3
CD4_T_m6
B_lympho
B_monocytes
B_neutrophils
B_leuko
total_B_m3
mat_B_m3
CD4_T_pre_exam
CD3_T_pre_exam
CD8_T_pre_exam
ASV_467 Legionella sp.
ASV_2791 Uncultured
ASV_741 Pseudomonas sp.
ASV_1157 Uncultured
ASV_617 Pseudomonas sp.
ASV_919 Veillonella sp.
ASV_2716 Legionella sp.
ASV_701 Pseudomonas sp.
ASV_762 Dialister sp.
ASV_1007 Dialister sp.
ASV_188 Streptococcus sp.
ASV_1115 Veillonella sp.
ASV_469 Veillonella sp.
ASV_117 Actinomyces naeslundii
ASV_2666 Rothia dentocariosa
ASV_34 Neisseria sp.
ASV_360 Corynebacterium sp.
ASV_16 Streptococcus sp.
ASV_2668 Moraxella nonliquefaciens
ASV_14 Corynebacterium_1 sp.
ASV_2667 Moraxella sp.
ASV_47 Rothia sp.
ASV_59 Streptococcus sp.
ASV_2768 Aeromonas sp.
B)
Cluster 1
Cluster 2
Cluster 3
CD3_T_m12
CD4_T_m6
Th17_m3
DonorMatch8
CD8_T_pre_exam
CD3_T_pre_exam
Amoxicillin1
CD4_T_pre_exam
K_pre_100
antibiotic_0
IDS
MDS
NHL
aGVHD_gradeII_IV
K_pre_70
IMD
week+3
week+2
Meropenem1
Ciprofloxacin1
Vancomycin1
PB
FLU_THIO
Donorhaploidentical_related
month+3
day0
month+1
ALL
Weight
TRM_00
pre_cond_start
Relapse
Survival_0
Karnofsky.bef.condt60
primary_diseaseCML
month+12
B_lympho
month+6
B_leuko
B_monocytes
K_d100_90
FLU_other
DonorMatch6
OL
B_neutrophils
BU_CY_VP16_MEL_combos
pre_exam
UCB
Meropenem0
TBI_CY_or_TBI_VP16
GVHD.prophylaxis7
bacterial_1
Ceftazidime1
primary_diseaseSAA
parasitic_infection1
Axis1 [9.13% variance]
Axis2 [5.27% variance]
−1
0
1
−2

Figure S6. Multivariate associations of the nasal microbiota with immune and clinical parameters in HSCT. A) Clustered image map (CIM) based on sparse partial least squares (sPLS) regression analysis dimensions 1, 2, and 3, displaying pairwise correlations >0.2/<-0.2 between nasal ASVs (bottom), and continuous immune and clinical parameters (right). Red indicated positive correlation, and blue indicates negative correlation, respectively. Based on the sPLS regression model, hierarchical clustering (clustering method: complete linkage, distance method: Pearson's correlation) was performed resulting in the three depicted clusters. B) Canonical correspondence analysis (CCpnA) relating nasal microbial abundances (circles) to continuous (arrows) and categorical (+) immune and clinical parameters. ASVs and variables with at least one correlation >0.2/<-0.2 in the sPLS analysis were included in the CCpnA. The triplot shows variables and ASVs with a score >0.3/<-0.3 on at least one the first three CCpnA axes, displayed on axis 1 versus 2 with samples depicted as triangles. The colored ellipses (depicted with 80% confidence interval) correspond to the clusters of ASVs identified by the sPLS-based hierarchical clustering. For visualization purposes, a focused section of the CCpnA triplot is shown. Antibiotics are indicated in blue font color. Abbreviations are described in Figures 6 and S5. Additional abbreviations: DonorMatch8, unrelated donor with 1 HLA mismatch; PB, peripheral blood.
